# Adaptation and validation of the Dutch version of the nasal obstruction symptom evaluation (NOSE) scale

**DOI:** 10.1007/s00405-017-4486-y

**Published:** 2017-03-03

**Authors:** Floris V. W. J. van Zijl, Reinier Timman, Frank R. Datema

**Affiliations:** 1 Department of Otolaryngology and Head and Neck Surgery, Erasmus University Medical Center, ‘s Gravendijkwal 230, P.O. 2040, 3000 CA Rotterdam, The Netherlands; 2 Department of Medical Psychology and Psychotherapy, Erasmus University Medical Center, Rotterdam, The Netherlands

**Keywords:** NOSE scale, Quality of life, Nasal obstruction, Validation, Dutch language

## Abstract

The nasal obstruction symptom evaluation (NOSE) scale is a validated disease-specific, self-completed questionnaire for the assessment of quality of life related to nasal obstruction. The aim of this study was to validate the Dutch (NL-NOSE) questionnaire. A prospective instrument validation study was performed in a tertiary academic referral center. Guidelines for the cross-cultural adaptation process from the original English language scale into a Dutch language version were followed. Patients undergoing functional septoplasty or septorhinoplasty and asymptomatic controls completed the questionnaire both before and 3 months after surgery to test reliability and validity. Additionally, we explored the possibility to reduce the NOSE scale even further using graded response models. 129 patients and 50 controls were included. Internal consistency (Cronbach’s alpha 0.82) and test–retest reliability (intraclass correlation coefficient 0.89) were good. The instrument showed excellent between-group discrimination (Mann–Whitney *U* = 85, *p* < 0.001) and high response sensitivity to change (Wilcoxon rank *p* < 0.001). The NL-NOSE correlated well with the score on a visual analog scale measuring the subjective sensation of nasal obstruction, with exception of item 4 (trouble sleeping). Item 4 provided the least information to the total scale and item 3 (trouble breathing through nose) the most, particularly in the postoperative group. The Dutch version of the NOSE (NL-NOSE) demonstrated satisfactory reliability and validity. We recommend the use of the NL-NOSE as a validated instrument to measure subjective severity of nasal obstruction in Dutch adult patients.

## Introduction

In 2004, Stewart et al. introduced the nasal obstruction symptom evaluation (NOSE) scale as a valid, reliable, and responsive self-report instrument to quantify the subjective burden related to nasal obstruction [1]. Patients are asked to answer five 5-point Likert Scale questions related to nasal obstruction resulting in a sumscore, ranging from 0 to 20, which is then multiplied by 5. The instrument is easy to complete with a minimal respondent burden, likely contributing to its global popularity in outcome research and surgical technique evaluation. This is illustrated by validated adaptations of the NOSE scale for the Spanish, Chinese, Italian, French, Greek, and Portuguese language [2–7]. Additionally, normative and abnormal value ranges for the NOSE scale have been outlined, allowing a more precise definition of treatment success and meaningful clinical changes of numerical scores [8]. The primary aim of this study was to translate and validate the NOSE scale instrument into the Dutch language.

An important remark when using (extensive) questionnaires to evaluate patient satisfaction, quality of life and change herein following medical treatment, is the influence of ‘respondent burden bias’ on given answers when questionnaires are too extensive. Although the NOSE scale is a relatively short questionnaire with only five items, the risk of inaccurate or incomplete answers might become important when the NOSE scale is offered to patients in addition to other questionnaires used for routine outcome monitoring (ROM). The secondary aim of this study was therefore to explore the possibility to reduce the NOSE scale into a more concise version including only the most indicative items.

## Materials and methods

This single-center instrument validation study consisted of a cross-cultural adaptation phase and a statistical validation phase. All data were prospectively collected between April 1, 2015 and September 1, 2016 at the department of otorhinolaryngology and head and neck surgery, and the department of urology of the academic Erasmus Medical Center, Rotterdam (the Netherlands). This study was approved by the Medical Ethics Committee of the Erasmus Medical Center, Rotterdam, the Netherlands, documented by Study Number MEC-2015-361.

### Phase 1: cross-cultural adaptation to the Dutch language

**Fig. 1:**
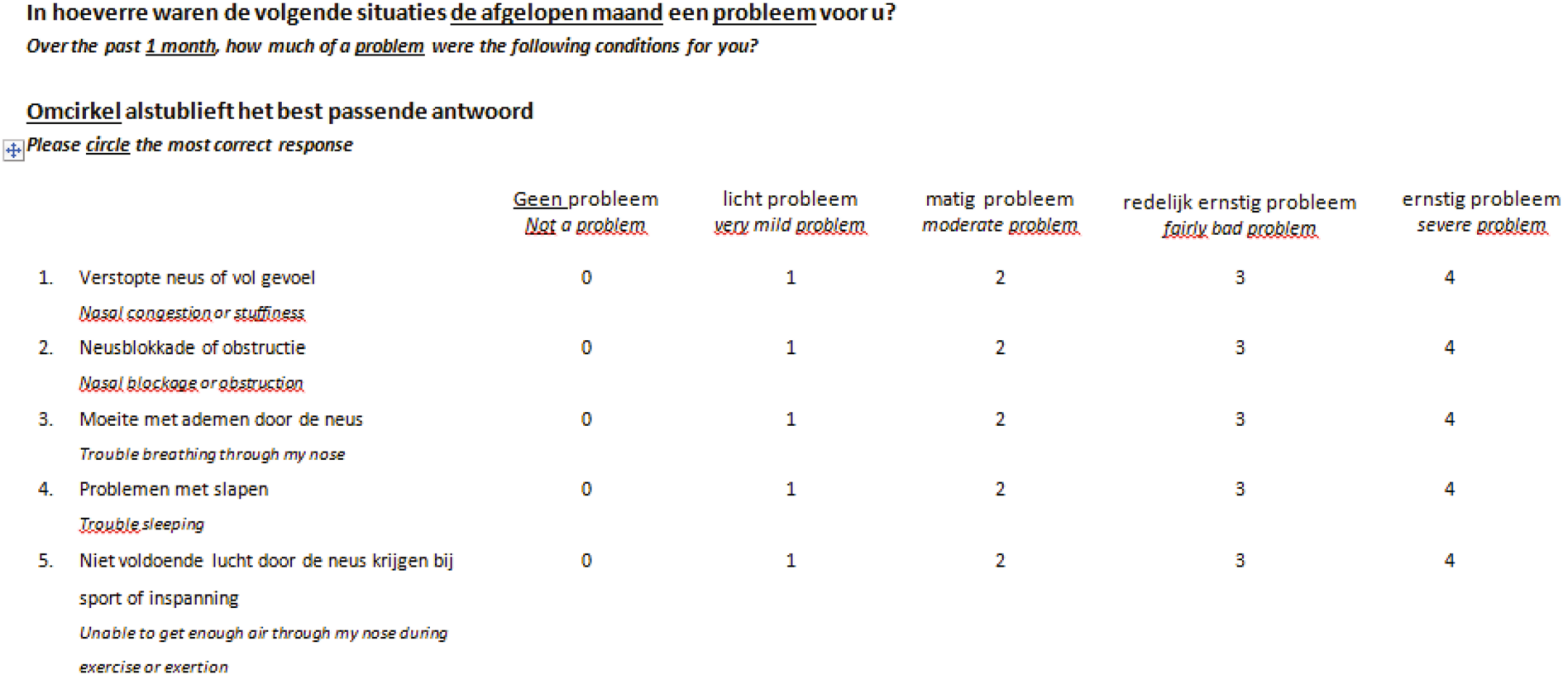
NL-NOSE adapted from the original NOSE scale (*italic*)

General accepted guidelines for the process of cross-cultural adaptation were followed [9]. Forward translation of the original NOSE questionnaire was performed by one bilingual Dutch-native otolaryngologist and one bilingual Dutch-native professional translator without medical background. The two bilingual investigators reconciled differences between the two forward translations and checked for semantic and conceptual equivalence, resulting in one single provisional Dutch translation of the NOSE scale. Two English-native translators without medical background then translated the provisional Dutch questionnaire back into the original language. These backward translations were compared with the original NOSE scale focusing on discrepancies and item content. The end result was a final version of the questionnaire (NL-NOSE, Fig. 1).

### Phase 2: NL-NOSE validation

#### Study populations

For this study, two separate populations were recruited prospectively. The first group included patients with nasal obstruction caused by a septal deviation and/or nasal valve insufficiencies. Patients were included when they were eligible for surgery, able to speak and read the Dutch language, and experienced nasal obstruction longer than 3 months, without a noticeable response to intranasal steroid treatment for a minimum of 4 weeks. We excluded patients younger than 18 years, patients with nasal obstruction related to mucosal disorders, craniofacial patients, or patients who had prior septoplasty/septorhinoplasty or turbinate surgery. The second group consisted of healthy asymptomatic controls recruited at the department of urology. Controls needed to be older than 18 years, be able to read and speak the Dutch language, and have no history of nasal obstruction and/or use of intranasal medication.

#### Methods and statistical analysis

Generally accepted quality criteria for validation were used as a guideline [10, 11]. Generally, in the various language NOSE validation studies, correlations of at least 0.40 with criterion measures were reported [2–6]. In order to detect a significant correlation coefficient of at least 0.40, we considered 50 cases as sufficient [12]. In cases where one out of five NL-NOSE items was missing, the total score was calculated from the mean of the completed items. If more than one item was missing, the case was excluded.

#### Internal consistency

Internal consistency was investigated using Cronbach’s alpha coefficient, which was considered fair when alpha was between 0.70 and 0.79, good between 0.80 and 0.89, and excellent above 0.90 [13]. Corrected item-total and inter-item correlations were tested using Spearman correlations. For assessment of unidimensionality, a confirmatory factor analysis (CFA) was performed in the preoperative, postoperative, and control groups. These CFAs tested single-factor models without allowing additional covariances between the items. All CFAs were applied using ordinary maximum likelihood that excludes cases with missing values. Standards for a good fit were derived from Brown [14]. The recommended index values are presented in Table 2.

#### Reproducibility

Test–retest reliability was investigated by administering a second NL-NOSE questionnaire 2 weeks after the first. This was carried out for the patient group only. Patients with any change in conservative treatment after completing the first questionnaire (medication, nasal steroids, other) or change of symptoms due to upper or lower airway infections were excluded for the assessment of test–retest reliability. Test–retest reliability was calculated using 2-way random average measures intraclass correlation coefficients (ICC), with a positive rating for reliability given at >0.70. Differences between responders and non-responders at the second test were analyzed with Mann–Whitney *U* tests and a *χ* ^2^ test.

#### Discriminant validity

Discriminant validity of the NL-NOSE was tested by comparison of the scores of the patient group with the asymptomatic control group with a Mann–Whitney *U* test, with a significant difference defined as *p* < 0.05.

#### Responsiveness

The response (sensitivity to change) was tested using a subgroup of patients who were asked to complete the NL-NOSE 3 months after surgery, assessed with the Wilcoxon rank test and calculation of the mean and inter-quartile range.

#### Construct validity

In the absence of an objective gold standard to quantify nasal patency, construct validity was assessed with a Spearman correlation test between NL-NOSE item scores and scores on a 100 mm Visual Analog Scale indicating nasal airway patency, ranging from 0 (very bad) to 10 (very good). Our predefined hypothesis reads “patients with higher NL-NOSE scores, indicating more subjective burden of nasal obstruction, will have higher scores on the nasal airway patency VAS.”

#### Graded response models

Although this study was not primarily set up to develop a shorter version of the NL-NOSE scale, an exploratory attempt was made to reduce the number of items. For this purpose, graded response models (GRMs) were fitted to assess the information provided by each individual item on the latent trait. We only utilized the samples for which the unidimensionality assumption was reasonably met. The likelihood method applied in these GRMs was mean and variance adaptive Gauss–Hermite quadrature.

CFA was performed with STATA version14.1 (StataCorp, College Station, TX 77845 USA); all other statistical analyses were performed with SPSS 21.0 (IBM SPSS, Armonk, NY, USA).

## Results

Based on inclusion and exclusion criteria, a total of 131 patients with an indication for functional septoplasty or septorhinoplasty and 51 asymptomatic controls completed the NL-NOSE questionnaire. 129 patients and 50 controls gave valid answers on at least 4 items. Of these 129 patients, 77 completed an additional retest questionnaire returned by postal mail, 47 did not respond, and 5 were excluded for retest analysis due to an unintended change in conservative treatment. No significant baseline differences were observed between responders and non-responders for the total NOSE scale (Mann–Whitney *U* = 1950, *p* = 0.80), age (*U* = 1925, *p* = 0.71), and gender (*χ* ^2^ = 0.043, *p* = 0.84). On November 1, 2016, 64 out of 129 patients were operated on, of whom 50 patients had sufficient follow-up time to complete an additional postoperative questionnaire 3 months after surgery. A total of 313 administrations had been performed, with a total of 13 missing values on individual items (0.83%). These missing values led to the exclusion of four cases (1.28%).

The patient population (*N* = 129) consisted of 82 males (63.6%), with a mean age of 34.6 ± 14.5 (range 17–74). Mean sumscore (0–100) was 70.5 ± 20.0 (SD). No significant correlations of the NL-NOSE with age were observed, and there were no significant differences between men and women (non-parametric tests, all *p* values >0.30).

### Internal consistency

Internal consistency of the NL-NOSE was high with a Cronbach’s alpha of 0.81 for the preoperative group (*N* = 129), and 0.91 in the postoperative group (*N* = 50). Item-total and inter-item correlations for both preoperative and postoperative measures are displayed in Table 1. In the preoperative group, all values were above 0.40 except for the correlation between items ‘trouble sleeping’ and ‘nasal blockage or obstruction’ (0.36), and the correlation between items ‘trouble sleeping’ and ‘unable to get enough air through my nose during exercise’ (0.32). The inter-item correlations within the control group were much lower, in particular for item 5, while the inter-item correlations for all participants combined were much higher. Relationships between the different variables were close with highly significant differences (*p* < 0.01) for all correlations.

**Table 1:**
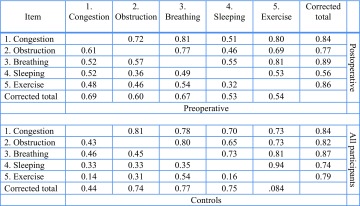
Inter-item and corrected item-total Spearman correlations of NL-NOSE

The confirmatory factor analysis in the preoperative group showed good indices for the CFI, TLI, and SRMR, but a lesser value for the RMSEA, although the chance that the RMSEA (pclose) is not significant is acceptable (Table 2, abbreviations enlisted), generally indicating that the unidimensionality assumption is reasonably met. In the postoperative group, all fit indices are excellent. The fit measures in the control group are poor, indicating that unidimensionality of the scale in this group is not satisfactorily established.

**Table 2:** Fit measures and confirmatory one-factor analysis

	Preoperative, *N* = 126	Postoperative, *N* = 50	Control, *N* = 50	All cases, *N* = 303	Recommended, Brown [14]
RMSEA	0.103	0.024	0.208	0.115	<0.05/<0.08*
pclose	0.112	0.471	0.015	0.008	>0.05
CFI	0.968	0.999	0.876	0.985	>0.95
TLI	0.935	0.998	0.752	0.971	>0.95
SRMR	0.033	0.022	0.070	0.014	<0.08

*RMSEA* root mean square error of approximation, *pclose* probability of RMSEA ≤0.05, *CFI* comparative fit index, *TLI* Tucker–Lewis index, *SRMR* standardized root mean squared residual

*<0.05 = good, <0.08 reasonable

### Reproducibility

Test–retest reliability (*N* = 77) was good with an intraclass correlation of 0.89 (*p* > 0.001).

### Control group and discriminant validity

**Fig. 2:**
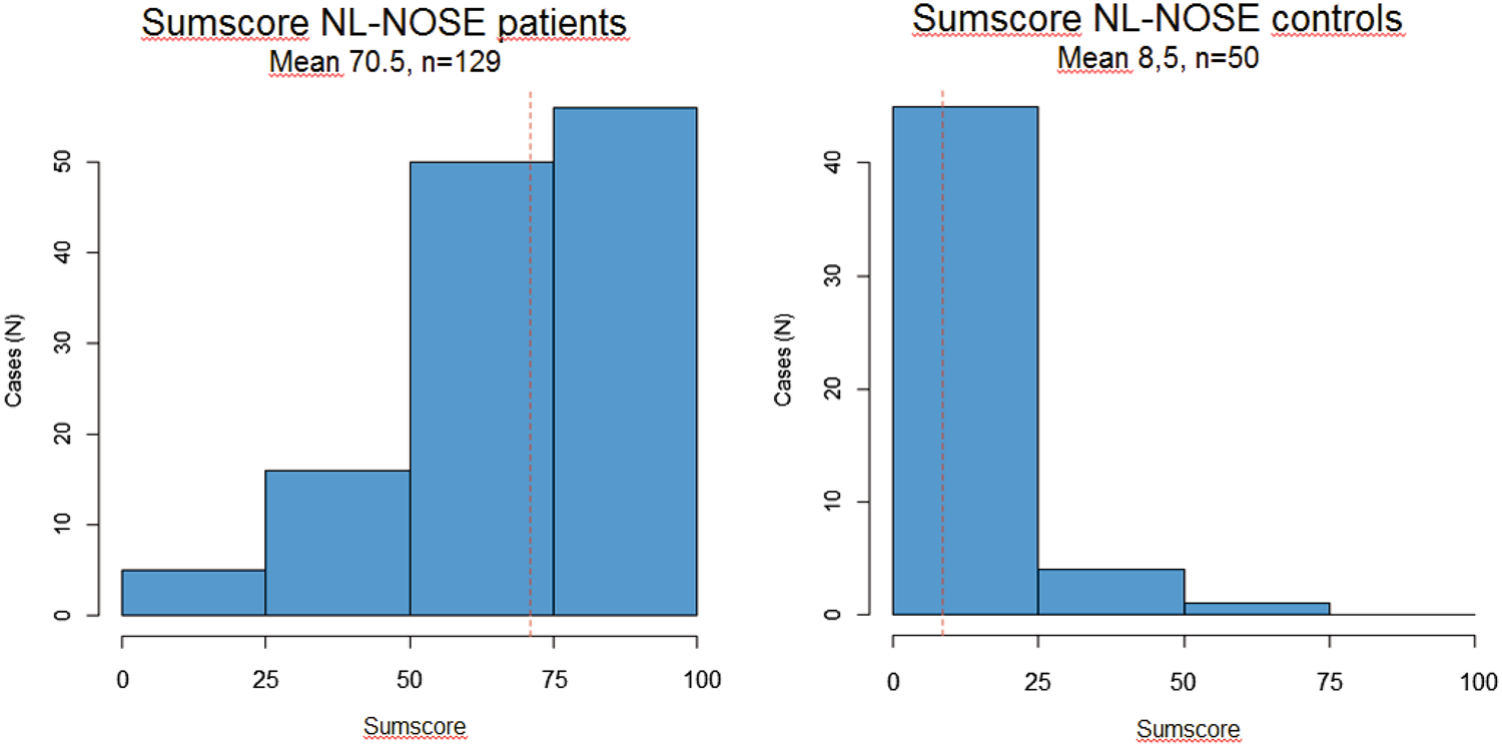
Sum scores of patients and controls

In the control group (*N* = 50), nineteen (38.0%) controls were male and the average age was 47.9 ± 16.8 (range 19–80). Mean sumscore was 8.5 with a standard deviation of 13.0 (Fig. 2). The NL-NOSE showed excellent discrimination between groups with a mean rank of 114.3 for patients and a mean rank of 27.2 for controls (Mann–Whitney *U* = 85, *p* < 0.001). Cronbach’s alpha in the control group was 0.79.

### Pre- and postoperative evaluation (responsiveness)

Patients that completed a questionnaire before and after surgery (*N* = 50) were all operated on by one author (FRD), performing either a septoplasty or (septo)rhinoplasty mainly aiming at restoring nasal patency. Postoperative mean sumscores were significantly lower compared to preoperative values (Wilcoxon rank *p* < 0.001). All but two patients had lower scores after the operation; these two patients reported no change. The magnitude of surgery effect was large; median sumscores dropped from 70.0 preoperatively to 20.0 postoperatively (median change 40.0, inter-quartile range 25–63).

### Correlation with VAS (construct validity)

Correlation of the mean VAS score (left and right) with the NL-NOSE sum score and individual items is shown in Table 3. Sum scores correlated well with the VAS, both for the symptomatic cohort pre- and postoperatively and for the control cohort, confirming our hypothesis. Regarding the individual items, only the item ‘trouble sleeping’ did not correlate well with VAS.

**Table 3:** Spearman correlations of NL-NOSE with VAS

Item	Preoperative, *N* = 129		Postoperative, *N* = 50		Control, *N* = 50	
	rho	*p*	rho	*p*	rho	*p*
1. Congestion	0.46	<0.001	0.66	<0.001	0.57	<0.001
2. Obstruction	0.43	<0.001	0.79	<0.001	0.50	<0.001
3. Breathing	0.40	<0.001	0.74	<0.001	0.45	0.001
4. Sleeping	0.12	0.165	0.36	0.002	0.20	0.169
5. Exercise	0.46	<0.001	0.67	<0.001	0.36	0.011
Total	0.44	<0.001	0.78	<0.001	0.65	<0.001

*rho* Spearman correlation

### Graded response models

**Fig. 3:**
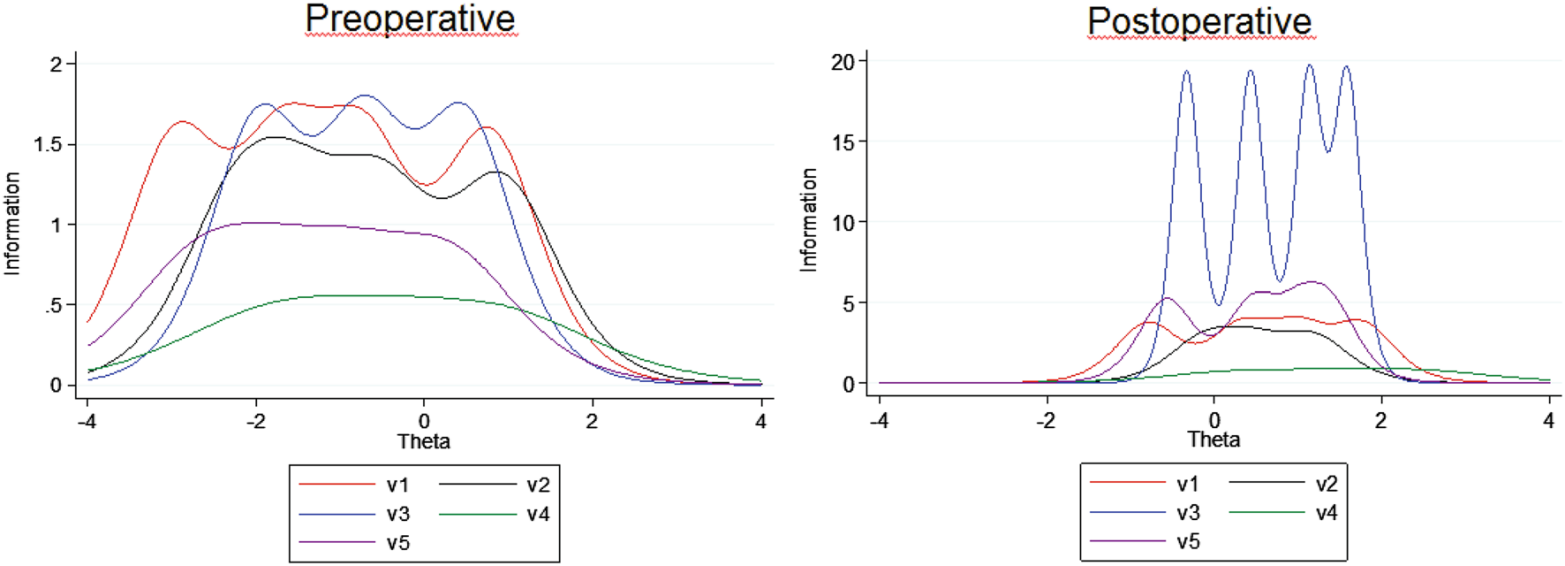
GRM item information functions for pre- and postoperative patients

We fitted in GRMs for the pre- and postoperative patients, as the unidimensionality assumption was reasonably met in these groups. It must be noted that these models are explorative, as Reise and Yu reported that a GRM can be estimated with 250 cases but a sample of at least 500 is advised [15]. Our preoperative group included only 131 cases for this analysis, and the postoperative group 51. In both samples, item 4 (trouble with sleeping) provided the least information to the total scale and item 3 the most, particularly in the postoperative group (Table 4; Fig. 3). These findings are confirmed with classical test theory CTT analyses; the Mann–Whitney *U* values are the largest for item 4 and the smallest for item 3 (Table 4). Mann–Whitney *Z*-values are the largest for item 3. These values for item 3 are about as large as for the total scale, suggesting that the total scale might not provide much more information than item 3.

**Table 4:** GRM item discrimination coefficients and differences in total NOSE scores between groups

	GRM item coefficient (95% CI)		Difference, pre- and postoperative		Difference, preoperative and controls	
	Preoperative, *N* = 131	Postoperative, *N* = 51	M-W, *U* value	M-W*, *Z* value	M-W *U* value	M-W*, *Z* value
1. Congestion	2.51 (1.55, 3.47)	3.87 (1.92, 5.81)	1102.0	7.09	317.5	9.63
2. Obstruction	2.26 (1.46, 3.06)	3.46 (1.70, 5.22)	938.0	7.50	364.0	9.44
3. Breathing	2.60 (1.56, 3.63)	8.80 (−1.50, 19.11)	807.5	8.07	150.0	10.18
4. Sleeping	1.33 (0.83, 1.84)	1.69 (0.70, 2.68)	1095.0	7.20	597.0	8.78
5. Exercise	1.82 (1.15, 2.49)	4.57 (2.09, 7.04)	116.0	7.15	241.0	9.10
Total			670.0	8.23	85.0	10.12

*M-W* Mann–Whitney *U* test

*All *p* values <0.001

## Discussion

Routine outcome measuring has become an important indicator for medical performance. Transparent outcome reports assist the patient in making an educated guess between health care providers as long as the instruments used are comparable. The use of patient-reported outcome measures, in the absence of globally accepted objective instruments, is feasible when the instruments used are validated. The NOSE scale is a validated, globally accepted instrument to quantify the burden related to nasal obstruction and change herein following nasal surgery. Cross-cultural adaptation of the NOSE scale makes it a valuable instrument allowing the comparison of outcome results between institutions and to organize multi-center studies. In that context, our need for a validated Dutch version of the NOSE scale became apparent.

Internal consistency measures the extent to which items in a questionnaire are correlated, which is an important measurement property for questionnaires that intend to measure a single underlying concept using multiple items such as the NOSE questionnaire [10]. We found a Cronbach’s alpha of 0.81 for the NL-NOSE, which is within accepted ranges and comparable to previously reported NOSE validation studies [1–5, 7]. When looking at the Cronbach’s alpha of the postoperative cohort, we found a value of 0.91. This is also reflected by Table 1, displaying that item correlations in the postoperative group are higher compared to the correlations of the preoperative group, and in Table 2 that the fit for a unidimensional model is better for the postoperative group.

The reproducibility of the NL-NOSE was confirmed by performing a test–retest, correlating initial test and subsequent retest scores. We found an intraclass correlation coefficient of 0.89, demonstrating that the questionnaire is stable over time. Normative data were generated by a cohort with no distinct complaints of nasal patency. This group scored a mean of 8.5 ± 13.0 compared to 70.5 ± 20.0 in the case cohort, suggesting that the NL-NOSE is a sensitive instrument to identify patients with nasal patency complaints. The correlations between the VAS and the total score of the NL-NOSE demonstrated good construct validity. We explored pre-, postoperative, and control group correlations, and found that the correlations with VAS in these separate patient groups were lower compared to the correlations documented in the Spanish and Italian validations studies [5, 6]. However, both the Spanish and Italian authors do not mention the composition of the group tested. When using the total cohort, we found higher correlations with VAS, comparable to those reported in the Italian study. These higher correlations are caused by the larger variance induced by the combination of low scoring controls and postoperative patients and high scoring preoperative patients for the total scale and their high respectively low VAS scores. Regarding the individual items, only the item ‘trouble sleeping’ did not correlate well with VAS, which is similar to the results of other validation studies. The GRM also pointed out that item 4 is not contributing very well to the total scale.

Perhaps most importantly, in line with other validation studies, the NL-NOSE demonstrated excellent responsiveness after surgery, indicating that the instrument is suitable for measuring treatment outcome. Median sumscores dropped from 70 to 20 after surgery, which is comparable to the systematic review of Rhee et al. reviewing NOSE scores of patients with nasal airway obstruction after septo(rhino)plasty with or without turbinate surgery [8]. The authors compiled scores and found a mean pretreatment score of 65 (standard deviation 22) and a mean posttreatment score of 23 (20). Furthermore, the authors found that that no individual study dropped less than 30 points, suggesting that a change of at least 30 may be considered a clinically meaningful measure of surgical success. Our results, with a median decrease of 40 points after surgery, therefore, confirm that the NL-NOSE is able to measure clinically meaningful success of nasal functional surgery.

We fitted GRMs in order to explore whether a more concise version of the NL-NOSE could be constructed. These models suggest that item 3 might be nearly as informative as the overall NL-NOSE sumscore. Future research pointed to this issue with larger study populations should be conducted to reach more definite conclusions.

A potential shortcoming of the study may be that the proportion of men is larger in the patient group compared to the control group. However, as we found no relation of the NL-NOSE with gender, we consider the influence of this difference to be minimal. Second, due to the lack of a Dutch questionnaire measuring nasal patency-specific quality of life that has been validated in functional (septo)rhinoplasty patients, we had no perfect gold standard to compare our results to. Instead, we chose to compare results to a nasal patency VAS score, for which our predefined hypothesis was met. Lastly, this is a single-center study performed in an academic hospital, potentially causing impaired generalizability or selection bias. In the original validation study of Stewart, however, the NOSE questionnaire revealed good measurement properties in a multi-center study with four academic hospitals, and Larrosa et al. included both a tertiary and regional center with comparable results [1, 6].

## Conclusion

This study was performed to adapt the NOSE questionnaire to the Dutch language. Satisfactory internal consistency, reliability, reproducibility, validity, and responsiveness were demonstrated. We recommend the use of the NL-NOSE to quantify the subjective burden related to nasal obstruction and change herein following surgical intervention in Dutch adults.
